# Supplementary material for: An updated systematic review and meta-analysis on efficacy of Sofosbuvir in treating hepatitis C-infected patients with advanced chronic kidney disease
Source: PLoS One. 2021 Feb 10;16(2):e0246594. doi: 10.1371/journal.pone.0246594 (PMC7875415; doi:10.1371/journal.pone.0246594)
Supplement: S1 File — (PDF) [file pone.0246594.s002.pdf]

Appendix. Description of search strategy in PubMed

We used controlled vocabulary (e.g. Mesh terms for MEDLINE) and also used PICO strategy, including items like, the population (Renal disease, hemodialysis, Hepatitis C), intervention (Sofosbuvir, direct acting antiviral).

(("Hepatitis C"[Mesh]) OR ("Hepatitis C, Chronic"[Mesh]))

OR

((Parenterally-Transmitted Non-A, Non-B Hepatitis [Title/Abstract]) OR (PT-NANBH [Title/Abstract]))

AND

(("Renal Dialysis"[Mesh]) OR ("Renal Replacement Therapy"[Mesh]) OR ("Renal Insufficiency"[Mesh]) OR ("Renal Insufficiency, Chronic"[Mesh]))

OR

((Renal Dialyses [Title/Abstract]) OR (Hemodialysis [Title/Abstract]) OR (Dialysis, Extracorporeal [Title/Abstract]) OR (Renal Replacement Therapies [Title/Abstract]) OR (Kidney Replacement Therapies [Title/Abstract]) OR (Kidney Replacement Therapy [Title/Abstract]) OR (Renal Insufficiencies [Title/Abstract]) OR (Kidney Insufficiency [Title/Abstract]) OR (Kidney Failure [Title/Abstract]) OR (Renal Failure [Title/Abstract]) OR (Acute Kidney Injury [Title/Abstract]) OR (Chronic Renal Insufficiencies [Title/Abstract]) OR (Chronic Kidney Insufficiency [Title/Abstract]) OR (Chronic Kidney Diseases [Title/Abstract]) OR (Chronic Renal Diseases [Title/Abstract])

AND

(("Sofosbuvir"[Mesh]) OR (PSI -7977[Title/Abstract])) OR (GS-7977 [Title/Abstract])) OR (Sovaldi [Title/Abstract]) OR (direct acting antivirals [Title/Abstract]) OR (NS5B polymerase inhibitors [Title/Abstract]) OR (direct acting nucleotide polymerase inhibitor [Title/Abstract]))

S1 Table. Detailed characteristics of included studies in meta-analysis of SVR12 rate in HCV-infected patients with advanced chronic kidney disease.

Firth author/year	Country	No. of patients	Recruitment period	Mean Age (SD)	Mean baseline HCV RNA (log 10 IU/ml)	Cirrhosis N (%)	No. of patient on dialysis / Total patient	Genotype (%)	Treatment strategy & duration of treatment	Dose (mg)	SVR 12 (%)	SVR 24 (%)	No. of patient with AE (%)
Taneja 2018 [24]	India	65	2016	49 (13)	1.65 × 106	21 (32.3%)	(54/65)	GT1 (64.6)<br>GT 2 (1.4)<br>GT 3: (34)	SOF + DCV<br>12 week: 44 PT*<br>24 week: 21 PT	200+60	100	_	Nausea: 5<br>Insomnia& headache: 4<br>pruritus: 1
Surendra 2018<br>[25]	India	19	2016	44	>800,000 IU/mL 12 (63%)<br>< 800,000 IU/mL 7 (37%)	0	(19/19)	GT 1a (63)<br>GT 1b (37)	SOF + LDV<br>12 week: 19 PT	400+90	100	_	Headache 1<br>Dizziness: 1
Manoj 2018<br>[26]	India	71	2015-2017	42	6.12 (3.0-7.84)	17 (23.9%)	(11/71)	GT 1 (62)<br>GT1a (54.9)<br>GT1b (7.1)<br>GT 3 (38)	SOF + RBV(24 w)<br>SOF + LDV(12 w)<br>SOF + DCV(12w)<br>12 week: 60 PT	400+200<br>400+90<br>400+60	100	98.5	Fatigue: 32<br>Headache: 16<br>Insomnia: 11<br>Nausea: 13

								GT3a (33.8)<br>GT3b (4.2)	24 week: 17 PT				Diarrhea: 15<br>Anemia: 21
Akhil 2018<br>[16]	India	22	2015-2016	49.8	2,642,495 IU/mL	0	(22/22)	GT 1: 63.6<br>GT 3: 27.2<br>GT 4: 9	SOF + RBV<br>SOF + LDV<br>SOF + DCV<br>12 week: 22 PT	400+200<br>400+90<br>400+60	100	_	Anemia: 9
Sperl 2017 [27]	Czech Republic	6	2015-2016	39	4,088,000 IU/mL	2 (33.3%)	(6/6)	GT 3a: 100	SOF + DCV<br>12 week: 6 PT	200+60	100	_	Diarrhea: 1
Dumortier 2017 [28]	France	50	2013-2015	60.5 (7.5)	2 603 063 ± 427 061	0	(35/50)	GT 1a(14)<br>GT 1b (42)<br>GT 2 (12)<br>GT 3 (10)<br>GT 4 (18)<br>GT 5 (4)	SOF + DCV<br>SOF + SMV<br>12 and 24 week	200+60<br>200+90	86	_	Severe anemia: 3<br>Headache: 16<br>Asthenia: 14<br>Diarrhea or nausea: 10 insomnia: 8
Cox-North 2017 [29]	USA	29	2014-2016	_	_	13<br>(44.8%)	(0/29)	GT 1 (3.4)<br>GT1a (48.6)<br>GT1b (20.7)<br>GT 2 (6.9)	SOF + LDV<br>SOF + LDV + RBV<br>SOF + DCV	400+90<br>400+90+200<br>400+60<br>400+60+200	97	_	anemia:3

								GT3 (17.2)<br>GT 6 (3.4)	SOF + DCV + RBV<br>12 and 24 week				
Choudhary 2017 [30]	India	16	2015-2016	45 (12)	7 (5–8)	2 (12.5%)	(16/16)	GT1 (68.7)<br>GT 3 (25)<br>GT4 (6.25)	PEGylated-IFN+SOF+RBV<br>SOF+DCV<br>12 week: 16 PT	135+400+200<br>400+60	80	_	severe thrombocytopenia: 1<br>fatigue: 2
Aggarwal 2017 [31]	USA	14	_	61 (4.9)	8375588.6<br>(12523305)	12 (85.7%)	(14/14)	GT 1 (60)<br>GT 2 (6.7)<br>GT 3 (20)<br>GT4 (13.3)	SOF + SMV<br>SOF + LDV<br>SOF + RBV<br>12 week: 9 PT<br>24 week: 5 PT	400+150<br>400+90<br>400+200	92.8	_	Headache: 1<br>Fatigue: 3<br>Acid reflux: 1
Agarwal 2017 [32]	India	62	2015-2016	33.8 (10.2)	$10^6(10^4-10^8)$	3 (4.8%)	(62/62)	GT1 (64.5)<br>GT 2 (1.6)<br>GT 3 (29)<br>GT 4 (3.2)<br>GT 6 (1.6)	SOF + RBV<br>SOF + DAC<br>12 week: 62 PT	400+200<br>400 + 60	95.2	_	Dyspepsia: 13<br>Tuberculosis: 4<br>bacterial pneumonia: 3
Singh<br>2016 [33]	USA	8	2014-2015	56.8<br>(20)	4.2 ± 6.9	3 (37%)	(8/8)	GT 1a (37)<br>GT 1b (37)<br>GT3 (12.5)	SOF + SMV<br>SOF + LDV<br>12 week: 8	400 + 150<br>400 + 90	100	_	nausea,vomiting:1<br>headache: 1<br>insomnia: 1

								GT4 (12.5)					
Saxena 2016 [34]	USA	17	_	≥65	1.6 (0.5–4.3)	-	-	GT 1a (45)<br>GT 1b (20)<br>GT 2 (17)<br>GT 3 (9)<br>GT 4 (2)	SOF+PEG+ RBV<br>SOF + RBV<br>SOF + SMV<br>SOF+SMV+ RBV<br>12 week: 73 PT	SOF: 400 mg<br>RBV: 800 mg (IQR: 400-1200)<br>SMV:150	88	_	
Nazario 2016 [35]	USA	17	_	57	HCV RNA level >800 000 IU/ml 13 (76%)	8 (47%)	(17/17)	GT 1a (76)	SOF + SMV	400+150<br>12 week: 17	100	_	Insomnia: 2<br>Nausea:1 Headache: 1<br>Anemia: 1
Desnoyer 2016 [36]	France	12	2014-2015	52	6.59	10 (83.4%)	(12/12)	GT 1a (25)<br>GT1b (41.6)<br>GT 1 (25)<br>GT 2 (8.3)	SOF + SMV<br>SOF + LDV<br>SOF + DCV<br>SOF + RBV	400 + 150<br>400 + 90<br>400 + 60<br>400 +200<br>12 week: 8 PT<br>24 week: 4 PT	83.3	41.6	Anemia:3<br>Headache:2
Beinhardt 2016 [37]	Austria	10	_	50.6 (10.9)	6.1 ± 0.8	4 (40%)	(10/10)	GT 1a (20)<br>GT 1b (40)<br>GT 3a (20)	SOF/SMV<br>SOF/DCV	400+150<br>400+60<br>12 week: 10 PT	96	96	Anemia:1<br>Thrombocytopenia:2<br>Hepatic encephalopathy: 1

								GT4a/c/d (10)<br>GT 4h: 0<br>GT1b/3a: 0					Pneumonia: 1<br>Peritonitis (Patient on PD): 1
Hundemer 2015 [38]	USA	6	2014	60 (14)	2,990,000 IU/mL	3(50%)	(6/6)	GT1 (100)	SOF+ SMV<br>SOF+ RBV<br>12 week: 4 PT<br>24 week: 2 PT	400+150<br>400+200	64	_	Anemia: 3<br>Leukopenia: 1<br>AKI (Lupus like immune complex diseases): 1
Goel 2018 [39]	India	41	2015-2017	41	5.90(4.1-9.9)	5 (12%)	(41/41)	GT 1 (42)<br>GT 3 (54)<br>GT 4 (4)	SOF+DCV<br>12 week: 35 PT<br>24 week: 6 PT	200+60	90.2	_	Acute mild pancreatitis: 1
Gupta 2018 [40]	India	7	2015-2016	48.8 ± 14.5	_	2 (28.6%)	(7/7)	GT1 (71.4)	SOF+RBV<br>SOF+DCV<br>12 week: 5 PT<br>24 week: 2 PT	200+200<br>200+60	100	_	Anemia: 1<br>Hypoglycemia: 1
Mehta 2018 [41]	India	38	2016	49.5	5.75 (5.05–6.36)	_	(26/26)	GT1a (42.1)<br>GT1b (57.9)	SOF+LDV<br>SOF+DCV<br>12 week: 38	400+90<br>400+60	100<br>92.3	_	_

Borgia 2019 [42]	Canada, the United Kingdom, Spain,<br>Israel, New Zealand, and Australia	59	2017-2018	60	5.8 (3.1–7.7)	17 (29%)	(59/59)	GT 1 (46)<br>GT 1a (25)<br>GT 1b (19)<br>GT 2 (12)<br>GT 3 (32)<br>GT 4 (7)<br>GT 6 (3)	SOF+ Velpatasvir<br>12 week: 42 PT<br>24 week: 17 PT	400+100	95	_	Headache: 10<br>Fatigue: 8<br>Nausea: 8<br>Vomiting: 8<br>Insomnia: 6
Poustchi 2020 [43]	Iran	103	2017-2018	50.3±13.5	1.2359	39 (37.9%)	(75/103)	G1 (51.5)<br>G2 (1.9)<br>G3 (26.2)<br>G4 (8.7)<br>Unknown(11.7)	SOF + DCV<br>12 week: 64 PT<br>24 week: 39 PT	400 + 200	100	_	Diarrhea: 3<br>Headache:4<br>Pruritus: 3<br>nausea: 3<br>somnolence:2
Eletreby 2020 [44]	Egypt	579	2014-2018	52	0.39 × 106	107 (11%)	10/579	NR	IFN + SOF + RBV<br>SOF + RBV<br>SOF + DAC<br>SOF + DAC + RBV<br>SOF + SIM<br>SOF + SIM + DAC	SOF: 400<br>RBV: 600	96.7	_	Anemia: 19<br>Leukopenia: 1<br>Thrombosis: 1

									12 week: 579 PT				
Debnath 2020 [45]	India	18	2017-2018	39.4 ± 8.3	2,35,000	0	18/18	G1: (66.7)<br>G2: (5.5)<br>G3: (22.3)<br>G5: (5.5)	SOF + LDV<br>SOF + DCV<br>12 week: 18	400 + 90<br>400 + 60	100	_	Nausea & dyspepsia: 4<br>Fatigue: 2<br>Headache: 1
Michels 2020 [46]	Brazil	241	2016-2017	60.7±10.4	NR	0	34/241	G1: (85.5)<br>G2: (2.5)<br>G3: (12)	SOF + DCV<br>SOF + SMV<br>SOF + RBV<br>12 week: 241	SOF: 400<br>RBV: 250	99.3	97.1	Anemia: 1
Cheema 2019 [47]	Pakistan	18	2017-2018	47.2±14.1	5.88 ± 6.0	4 (22.2%)	18/18	G1: (33.4)<br>G3: (66.7)	SOF + DCV<br>12 week: 14<br>24 week: 4	400 + 60	83.3	83.3	NR
Mandhwani 2020 [48]	Pakistan	133	2016-2018	31.9 ± 9.8	NR	0	133/133	G1: 67 (50.3)<br>G2: (0.7)<br>G3: (42.9)<br>G4: (1.48)	SOF+DAC<br>12 weeks: 133	400 + 60	96.9	_	Anemia: 58<br>Seizure: 1
Seo 2020 [49]	Korea	9	2017-2018	59.9	NR	2 (22.2%)	9/9	NR	SOF + RBV<br>12 weeks: 9	400 + (100 to 200)	100	_	Anemia: 5<br>Insomnia: 1

													Nausea: 1<br>Itching: 2<br>Fatigue: 2

†SVR=Sustained viralogical response; ‡SD=Standard deviation; §PT=Patient; ¶GT=Genotype

S2 Table. Severe adverse event, discontinuation rate and mortality rate.

Study	No. of patient	No. of patient with SAE (%)	Description of SAE	Number of discontinuations	Number/description of Mortality rate
Beinhardt 2016	10	5 (50%)	recurring peritonitis, renal anemia, graft failure after orthotropic liver transplantation (OLT), cirrhosis due to HCV recurrence after OLT, pneumonia	_	_
Hundemer 2015	6	_	_	1 (0.16)	_
Goel 2018	41	2 (4.9%)	acute mild pancreatitis after renal transplantation: 1<br>worsening of ascites: 1	2 (0.05)	3 (0.07)<br>3 PT: Not due to treatment
Gupta 2018	7	_	_	1 (0.14)	1 (0.14)<br>1 PT: NR*
Borgia 2019	59	11 (19%)	increased daytime urinary frequency<br>renal colic	1 (0.02)	2 (0.03)<br>1 PT: from suicide<br>1 PT: died of metastatic lung cancer
Aggarwal 2017	14	_	_	2 (0.14)	1 (0.07)

					1 PT: NR
Cox-North 2017	29	1 (3.4%)	cardiac event (unable to draw any conclusions about the safety of SOF regimens in those with underlying cardiac disease): 1	_	1 (0.03)<br>1 PT: myocardial infraction
Dumortier 2017	50	_	_	_	2 (0.04)<br>2 PT: liver failure<br>1 PT: unknown reasons
Poustchi 2020	103	_	NR	_	3 (0.03)<br>1 PT: Bladder cancer<br>1 PT: myocardial infarction<br>1 PT: pulmonary edema
Eletreby 2019	579	4 (0.69)	Hematological complications: 2<br>Worsening renal functions: 2	2 (0.003)	1 (0.002)<br>1 PT: unknown reasons
Debnath 2020	18	_	_	_	2 (0.11)

					1 PT: due to inadequate hemodialysis<br>1 PT: unknown reasons
Michels 2020	241	_	_	1 (0.03)	_
Cheema 2019	18	_	_	3 (0.17)	_
Surendra 2018	19	-	-	-	2 (0.11)<br>2 PT: inadequate dialysis

†SAE=Severe adverse event; ‡NR=Not reported; §PT=Patient; ¶OLT= Orthotropic liver transplantation

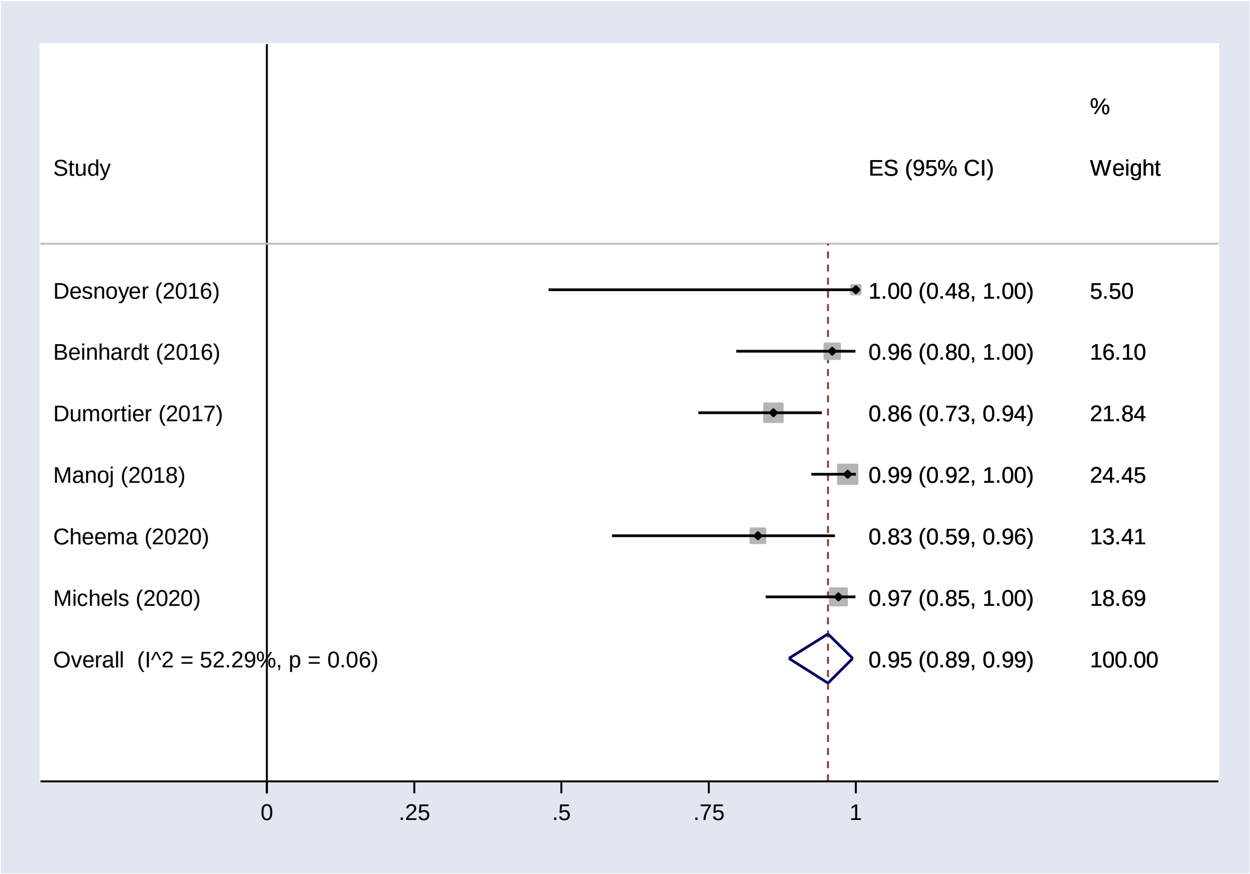

S1 Fig. Forest plot of pooled SVR24 rate in HCV-infected patients with advanced chronic kidney disease

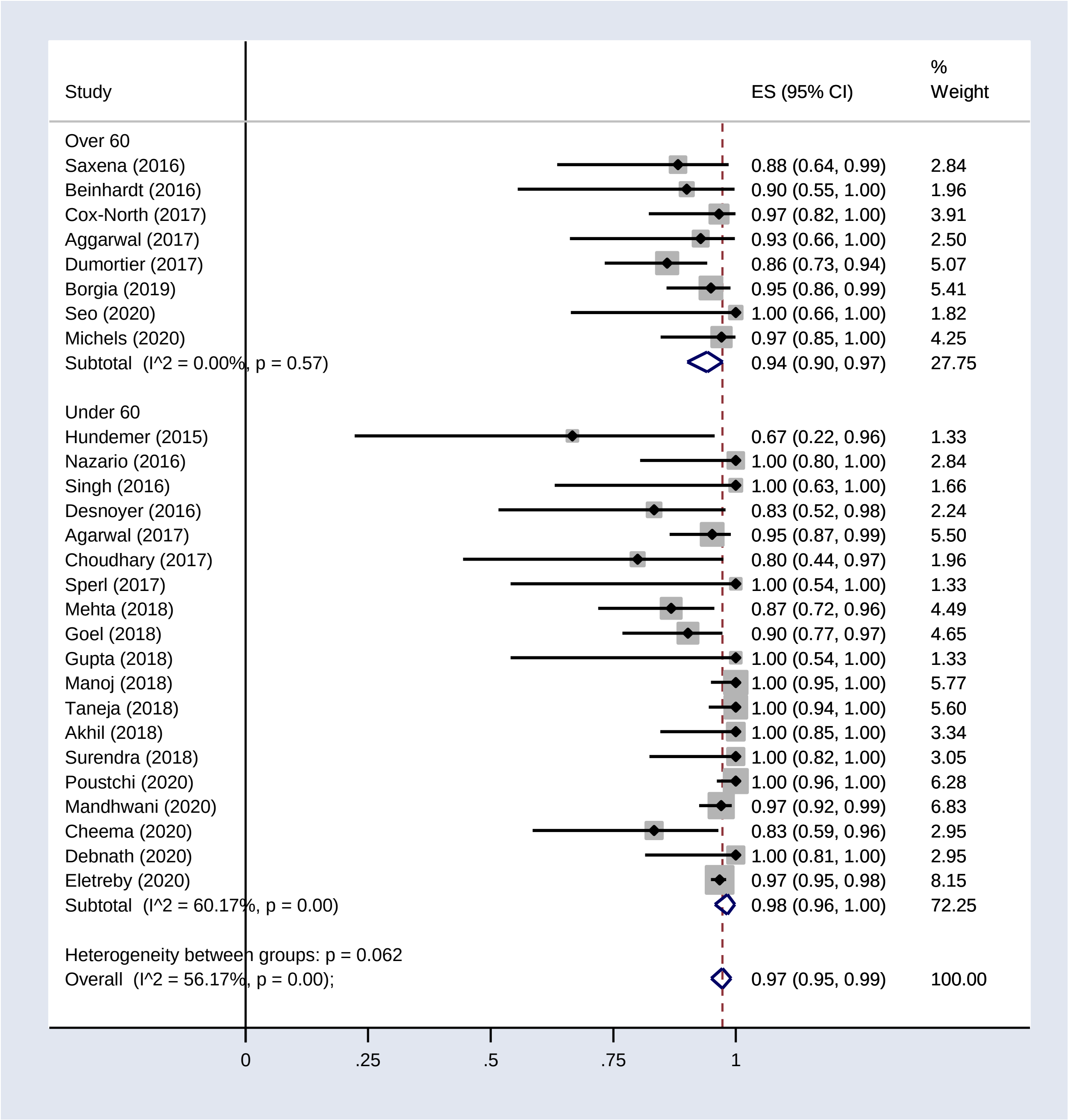

S2 Fig. Forest plot of pooled SVR12 rate in HCV-infected patients with advanced chronic kidney disease stratified by the age group

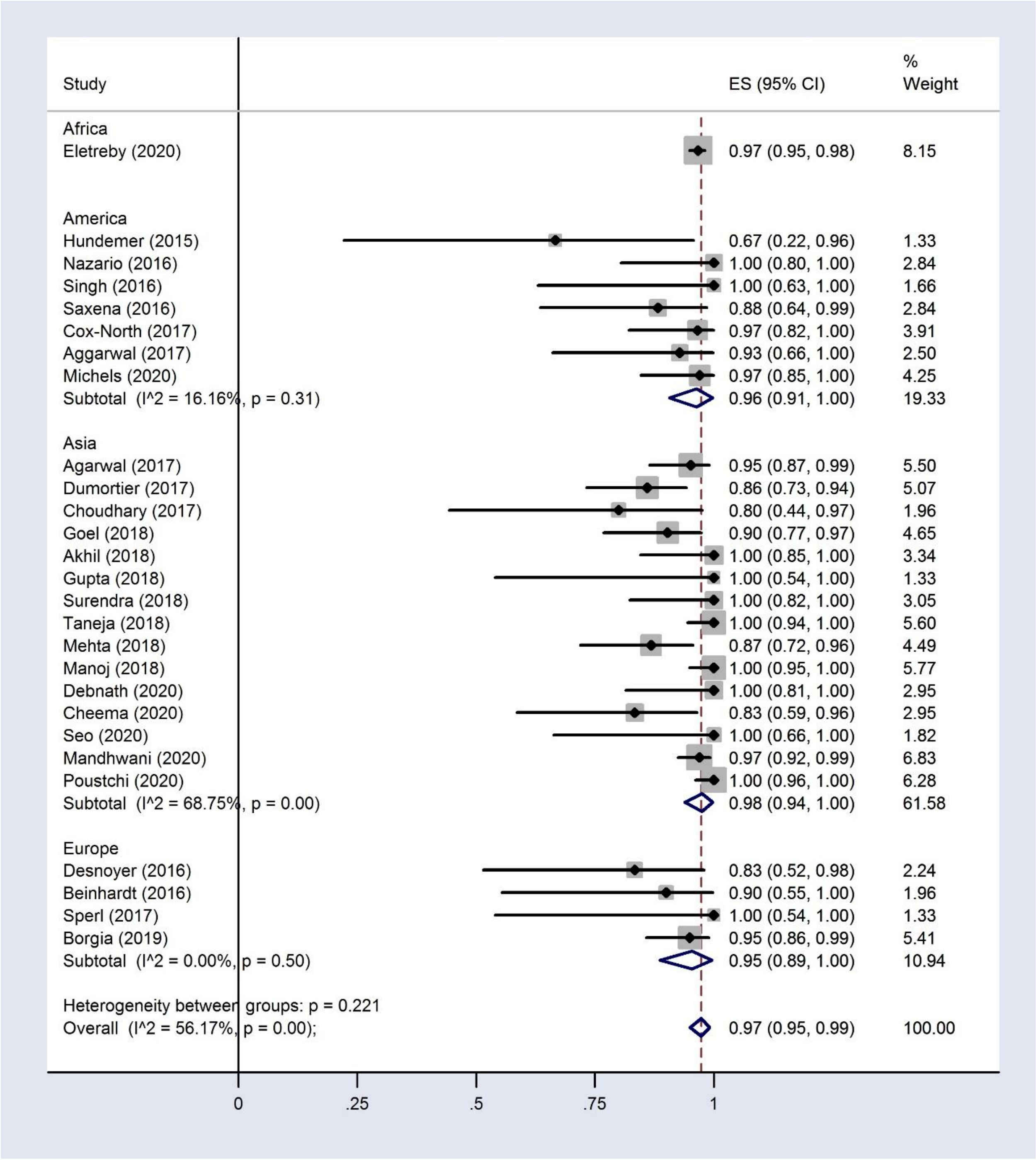

S3 Fig. Forest plot of pooled SVR12 rate in HCV-infected patients with advanced chronic kidney disease by the region of study

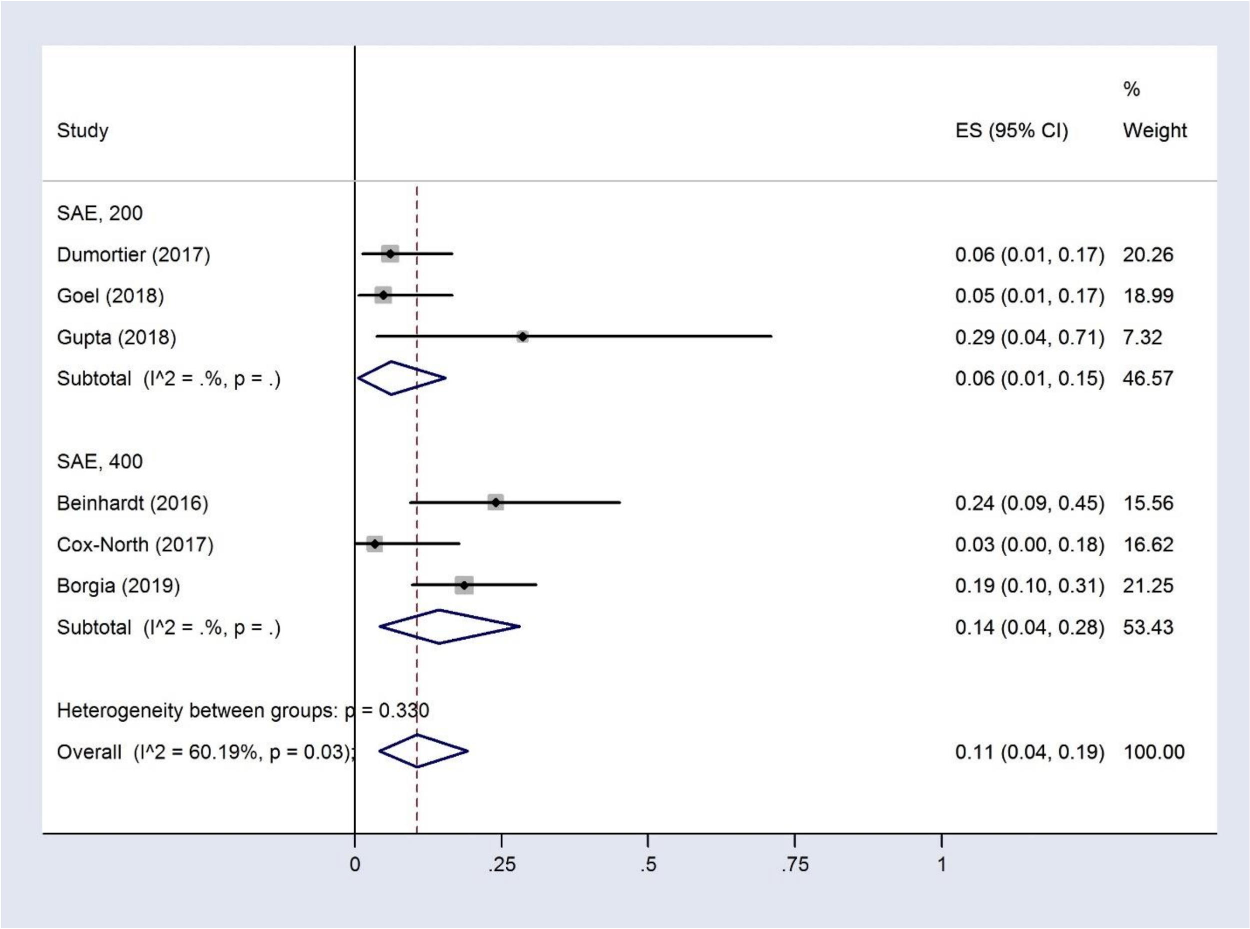

S4 Fig. The forest plot for incidence of severe adverse events among included studies

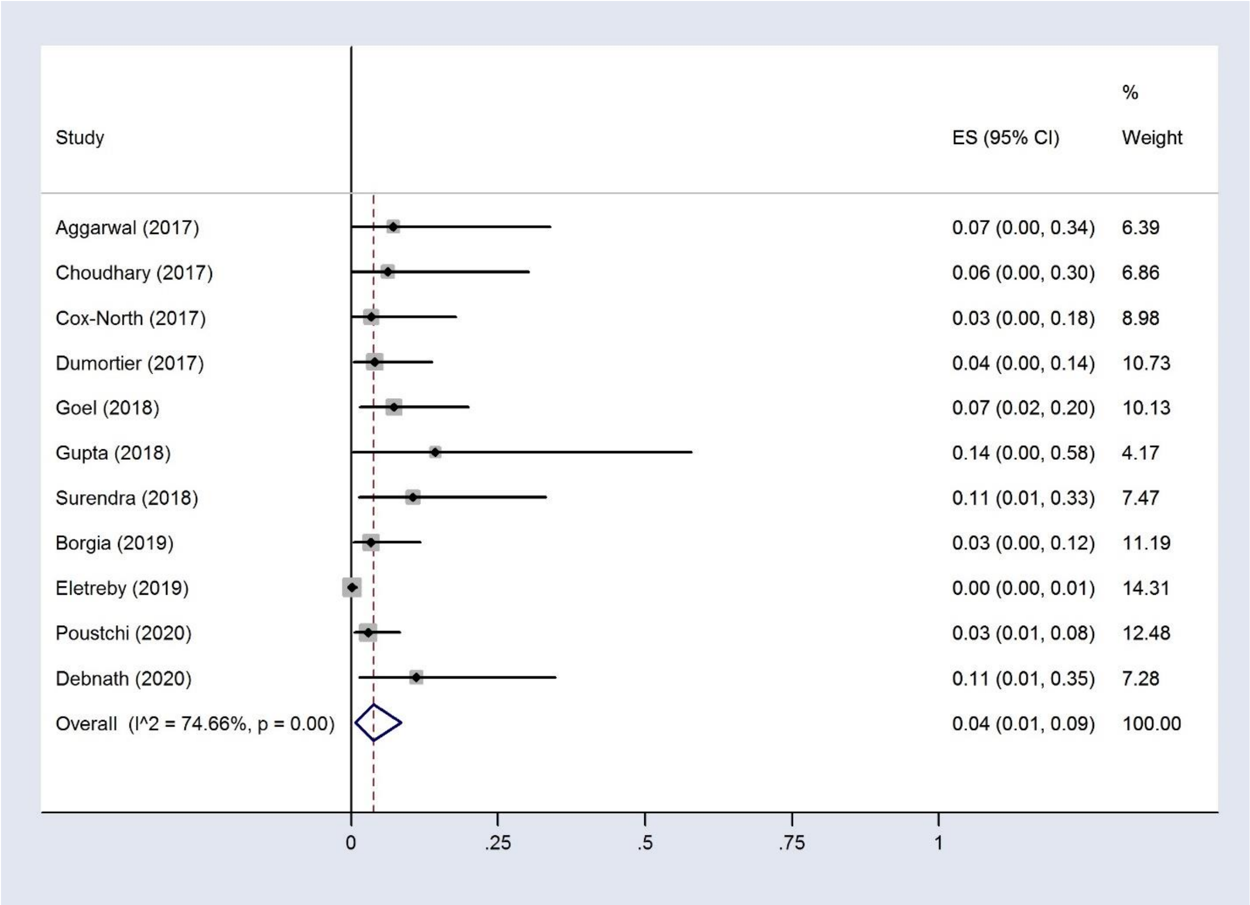

S5 Fig. The forest plot of pooled mortality rate among included studies

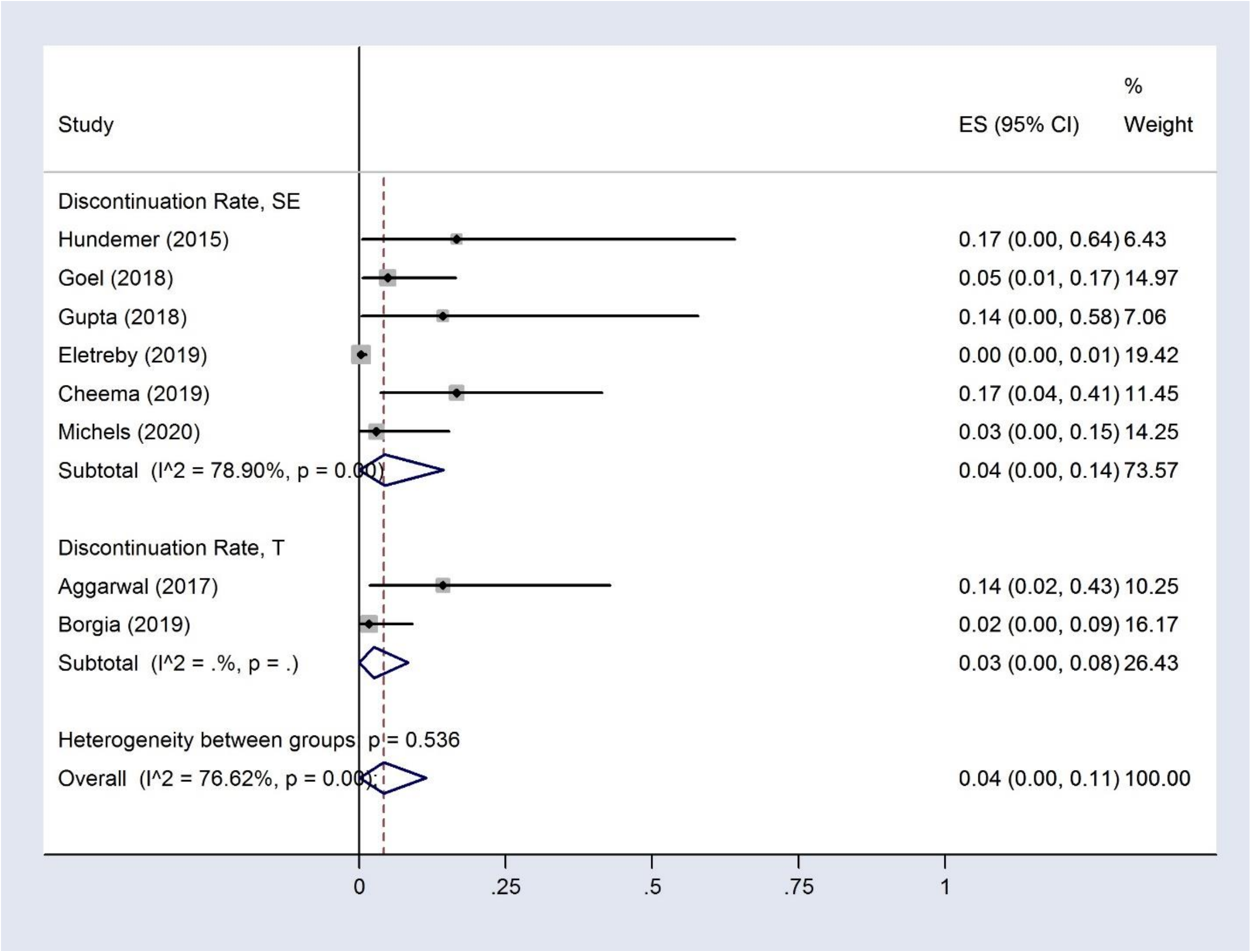

S6 Fig. The forest plot for incidence of discontinuation rate among included studies
