# Supplementary material for: An updated systematic review and meta-analysis on efficacy of Sofosbuvir in treating hepatitis C-infected patients with advanced chronic kidney disease
Source: PLoS One. 2021 Feb 10;16(2):e0246594. doi: 10.1371/journal.pone.0246594 (PMC7875415; doi:10.1371/journal.pone.0246594)
Supplement: Attachment — Submitted filename: Response_to_Reviewers.docx [file pone.0246594.s003.docx]

Dear Dr. Chen-Hua Liu,

**Thank you very much indeed for considering our manuscript for publication in PLOS One and your detailed comments. We entirely repeated the analyses based on the comments of respected reviewers. Please find our point-by-point response to your comments and the comments of the respected reviewers.**

1. Please ensure that your manuscript meets PLOS ONE's style requirements, including those for file naming. The PLOS ONE style templates can be found at

https://journals.plos.org/plosone/s/file?id=wjVg/PLOSOne_formatting_sample_main_body.pdf and

https://journals.plos.org/plosone/s/file?id=ba62/PLOSOne_formatting_sample_title_authors_affiliations.pdf

**Response: We modified our manuscript according to the requirements of the journal.**

2. Please include the following publication in your introduction and discuss what your research contributes in light of this recent published work: https://www.ncbi.nlm.nih.gov/pmc/articles/PMC7459301/

**Response: Thanks for this very valuable comment. We included this publication and added the following paragraph to the introduction section of the manuscript:**

**“In a recent systematic review, the safety and efficacy of Sofosbuvir has been explored among patients on HD.[20] The results are quite similar to the results of the current study. However, there is a very important difference between the two studies. In our study, we included patients with eGFR<30 ml/min/1.73 m2 and our search was not confined to patients on HD. This difference is quite important as the risk of accumulation of toxic metabolites in advanced CKD may be even higher than patients on HD. We also excluded studies on acute HCV and studies on patients with HBV and HIV co-infection. We have also conducted subgroup and sensitivity analyses”.**

3. In the Methods, please state the justification the restriction of the timeline to January 2013 onwards.

**Response: We mentioned in the methods section that we restricted the timeline to January 2013 as Sofosbuvir was approved for medical use in that year.**

Reviewer comments:

Reviewer #1: Dear Dr. Sepanlou,
Thank you for your effort on the field about effectiveness and safety of sofosbuvir-based regimen in ESRD patients. Several questions should be clarified.

1. Based on our knowledge, ESRD is defined as patient's eGFR less than 15 ml/min or receiving renal replacement therapy. However, in this study, ESRD was defined as eGFR less than 30 ml/min. You may revise the title and manuscript to "advanced CKD stage" rather than ESRD alone.

**Response: Thanks for this valuable comment. We replaced ESRD with “advanced CKD” in the title and throughout the manuscript.**

2. Line 142, the total patient number is 1571 less than the sum of male and female patients. There would be a mistake in recording patient numbers.

**Response: We repeated all analyses based on your valuable comments. We corrected the numbers: “The total sample size of 27 included articles amounted to 1464 patients, among whom 809 were males and 655 were females.”**

3. Table 1. reference number [26] and [27], the patients were acute infected by HCV virus and the treatment duration was different. Dose these two studies be suitable for enrollment?

**Response: Thanks for this valuable comment. We dropped these two studies on acute HCV patients and repeated all analyses.**

4. Table 1. reference number [30] Saxena, the eGFR of these 73 renal-impaired patients was less than 45 ml/min. Dose these patient met the inclusion criteria?

**Response: Thanks for this very valuable comment. You are quite right and we are sorry for this mistake. The study by Saxena et al is mainly conducted on 73 patients with GFR <45 ml/min. But in their subgroup analyses, they have reported the SVR among 17 patients with GFR < 30 ml/min as well. But details about these 17 patients are not reported. Therefore, we used this study only in estimating the overall SVR 12 reported for 17 patients but we dropped it from all other sub-group analyses.**

5. Table 1. reference number [31] Eletreby, the SVR of patients with renal impairment is 96.7% but the SVR of patients with ESRD is 80%. However, the SVR of ESRD patients seems not be enrolled in this meta-analysis 80% is patients on dialysis.

**Response: We used the SVR of 96.7% among patients with GFR <30 ml/min in our meta-analysis. We haven’t done a meta-analysis exclusively on patients on hemodialysis.**

6. Table 1, reference number [34] Manoj, the patient numbers and percentage of cirrhosis were not compatible with original study, please confirm the accuracy of data extraction.

**Response: Thanks very much indeed for this really valuable comment. We corrected the entire table 1.**

7. Please reconfirm the accuracy of patient numbers, SVR rate, the definition of CKD in all the enrolled studies.

**Response: We reviewed and revised the entire table 1.**

Reviewer #2: The authors did an updated systemic review and meta-analysis on the effectiveness of sofosbuvir (SOF)-based regimens in treating hepatitis C virus (HCV)-infected patients with end-stage renal disease (ESRD) from 29 published articles including 1571 subjects. They found that the pooled sustained virologic response (SVR) rates were 97% and 98% 12 and 24 weeks after cessation of antiviral therapies, respectively. Age, cirrhosis, and dose-reduction of SOF did not affect the treatment efficacy while adding on ribavirin (RBV) was associated with a higher incidence of treatment discontinuation and adverse events especially anemia. Though not a novel study, this manuscript has its scientific value in providing healthcare provider guidance in treating chronic HCV-infected patients who have end-stage renal disease. However, several issues deserve clarification or amendment in its current version.

1. Give necessary references to the following sentence “In earlier studies, PEGylated interferon (Peg-IFN) monotherapy…in HCV-infected ESRD patients.” in the second paragraph of the introduction.

**Response: Thanks indeed for this valuable comment. We added the following reference:**

**“KDIGO clinical practice guidelines for the prevention, diagnosis, evaluation, and treatment of hepatitis C in chronic kidney disease. Kidney international Supplement. 2008;(109):S1-99. Epub 2008/05/03. doi: 10.1038/ki.2008.81. PubMed PMID: 18382440.”**

2. Change ribavirin abbreviation to “RBV” instead of “rib” in the context.

**Response: We used “RBV” throughout the manuscript as you kindly mentioned.**

3. Did HBV co-infection exclude from the enrolled studies?

**Response: Yes, we excluded studies on patients with HBV or HIV co-infection and we mentioned it in introduction and methods sections.**

4. Explain why the number of included studies was greater than 29 in the subgroup analysis table 2?

**Response: The number of included studies was greater than 29 (currently 27 studies as we dropped two studies on patients with acute HCV) in subgroups because in some studies, more than one subgroup analysis has been reported. For example, in the study by Desnoyer et al, the SVR for treatment strategies SOF+DCV, SOF+LDV, SOF+RBV, and SOF+SMV have been separately reported. So the results of the study have been reported in all subgroups. Another example is the SVR reported based on whether the treatment was only SOF based or was combined with RBV. In a number of studies, both of these two regimens have been separately reported.**

5. Polish the language for the context such as correct “Sustained viralogical response” to “Sustained virologic response” in Table 2 footnote, and remove unnecessary capital to medication names.

**Response: Thanks indeed for this comment. We changed the “Sustained viralogical response” to “Sustained virologic response” throughout the manuscript.**

6. In ‘Sensitivity analysis’ please explain why references 30 and 31 were excluded for SVR calculation. The 2nd & 3rd sentences under this subject “The highest and lowest SVR12…respectively. So the pooled SVR12 rate was stable across studies.” were poorly understood.

**Response: As we dropped two studies on acute HCV patients based on the comments of reviewer #1, there are 27 studies in this meta-analysis. In the sensitivity analysis we excluded studies one by one to estimate the effect of their exclusion on the pooled analysis on the remaining 26 studies. When we excluded the study of Durmontier et al (in the revised version of this manuscript), the pooled SVR on the remaining 26 studies was highest (98.9%) and when we excluded the study by Poustchi et al, the pooled SVR on the remaining 26 studies was lowest (95.3%). When we excluded other studies one by one, the SVR on remaining 26 studies lied between 95.3% and 98.9%. We hope this explanation is adequate.**

7. Address the causes of the high incidence of SAE in SOF-based regimen to HCV-infected ESRD patients since the potential harm of its inactive metabolite GS-331007 to these subjects is the most concern issue by healthcare providers.

**Response: Thanks for this comment. The main message of this study is the low SAE in SOF-based regimens which confirms the utility, the safety, and the effectiveness of this treatment strategy and can’t be a concern for healthcare providers. This point is mentioned in the manuscript.**

8. Give proper author names and published year in Figure 2.

**Response: Thanks for this comment. All figures were re-drawn and corrected.**
