# Supplementary material for: An updated systematic review and meta-analysis on efficacy of Sofosbuvir in treating hepatitis C-infected patients with advanced chronic kidney disease
Source: PLoS One. 2021 Feb 10;16(2):e0246594. doi: 10.1371/journal.pone.0246594 (PMC7875415; doi:10.1371/journal.pone.0246594)
Supplement: Attachment — Submitted filename: Response_to_Reviewers.docx [file pone.0246594.s004.docx]

Dear Dr. Chen-Hua Liu,

**Thank you very much indeed for considering our manuscript for publication in PLOS One and your detailed comments. Please find our point-by-point response to the comments of the respected reviewers.**

Reviewer #1: Dear Dr. Sepanlou,
Thank you for revised the manuscript. Several issues should be clarified.

1. Line 101 ~ 107: Please revise English. Please do not use "Inclusion criteria :" For example : "All studies published in full English reporting the effectiveness and safety profiles of sofosbuvir-based regimen for treatment-naive chronic HCV-infected patients with advanced chronic kidney disease would be eligible." This would be more fluent and easy to be understood.

**Response: Thanks indeed for this comment. We modified the text accordingly and used the sentence that you kindly suggested, though the sentence is rather long.**

2.Line 168~171, Please well clarify the result of your subgroup analysis rather than using the Fig demonstrates ...

**Response: The following sections were added:**

**“Results sub-grouped by region of study are presented in S3 Fig showing lower SVR12 rates in Europe [95% (89-100)]. Fig 5 demonstrates the pooled SVR12 rate sub-grouped based on being treated only by Sofobuvir [99% (98-100)] or by its combination with RBV [99% (95-100)]. We additionally defined subgroups by treatment strategy such as the result of pooled SVR12 rate in 19 studies in which Sofosbuvir was used in combination with Daclatasvir [97% (94-99)],** [**Simeprevir**](https://www.google.com/search?sa=X&biw=1366&bih=657&sxsrf=ACYBGNRW_MAMV6yo0ur9xhDdND9vern3dg:1569231804458&q=Simeprevir&stick=H4sIAAAAAAAAAOPgE-LVT9c3NEw2qCg2zEvOUeLSz9U3yEsxNjZM0RLOTrbST87Pzc3PsyrOTEktT6wsXsTKFZyZm1pQlFqWWQQAudAQfz8AAAA&ved=2ahUKEwiTi5mC1ObkAhXOyKQKHUhLD6wQmxMoAzAWegQIDBAY) **[99% (94-100)], and Ledipasvir [100% (100-100)], which are reported in Fig 6. Table 2 demonstrates the summary of all sub-group analyses.”**

3.Line 197~200, Please clarify what you want to tell us. You want to illustrate age and country were associated with SVR12 rate in univariate analysis and none were found to be associated with SVR12 in multi-variate analysis?

**Response: Yes, we want to say the exact points you mention here. We corrected the sentence accordingly:**

**“There was a significant association between age (P=0.03, β=13.4) and country (P=0.02, β=-18.1) and the pooled SVR12 rate in the univariable model, which disappeared in the multivariable model. None of the variables of cirrhosis diagnosis, treatment strategy, and dose of treatment had significant association with the pooled SVR12 rate, neither in the univariable model nor in the multivariable meta-regression model.”**

4.Sensitivity analysis: I still could not understand what this mean. Is there any statistical evidence that sensitivity test is done by excluding the lowest and highest extreme values of enrolled studies. The result of pooled SVR12 rate remain around 90~98% after excluding the extreme value didn't mean the meta-analysis is more reliable.

**Response: Sensitivity analyses are integral components of meta-analysis. Sensitivity analysis may explore the impact of excluding or including studies in meta-analysis based on sample size or variance (and not extreme values). If results remain consistent across different analyses, the results can be considered robust as even with different decisions to include or exclude the studies, they remain the same / similar. If the results differ across sensitivity analyses, this is an indication that the result may need to be interpreted with caution.  It is very important to note that we haven’t excluded the extreme values. You can notice that Dumortier and Poustchi have not reported extreme values. We excluded each one of the studies out of the entire 27 studies in each step and performed the analyses on the remaining 26 studies. So we repeated 27 sensitivity analyses and we made 27 different estimates of SVR12 upon exclusion of each study from the list. The highest and the lowest estimates across these 27 analyses were reported in the text upon exclusion of studies by Dumortier and Poustchi respectively. Here, the analyses show that the results of these 27 sensitivity analyses are consistent and within the range of the overall SVR12 across all 27 studies. Therefore, our results are actually robust and don’t depend on the choice of included studies. We hope these explanations are adequate. We added the following section to the manuscript:**

**“To assess the effect of all studies on the pooled SVR12 rate, we used sensitivity analyses. In each step, we excluded one of the total of 27 studies and calculated the pooled SVR12 rate across the remaining 26 studies. Thus, we performed 27 sensitivity analyses and we made 27 different estimates of SVR12 upon exclusion of each study in each step. The highest and lowest estimates after excluding the studies by Dumortier [28] and Poustchi [43] were 98.9% (90.8-100) and 95.3% (91.1-100) respectively. So the pooled SVR12 rate was stable across studies. Our results are actually robust and don’t depend on the choice of included studies.”**

5. English editing of this manuscript would be necessary and helpful.

**Response: Thanks for this very important comment. We edited the entire manuscript. We made a major change and used the term “efficacy” instead of “effectiveness” in the title and throughout the entire text of the manuscript.**

Reviewer #2: The authors revised the manuscript according to my comments point-by-point, I have no further comments.

**Response: Thanks indeed for your positive feedback.**
